# Review of Advanced Hydrogel-Based Cell Encapsulation Systems for Insulin Delivery in Type 1 Diabetes Mellitus

**DOI:** 10.3390/pharmaceutics11110597

**Published:** 2019-11-12

**Authors:** Albert Espona-Noguera, Jesús Ciriza, Alberto Cañibano-Hernández, Gorka Orive, Rosa María Hernández, Laura Saenz del Burgo, Jose Luis Pedraz

**Affiliations:** 1 NanoBioCel Group, Laboratory of Pharmaceutics, School of Pharmacy, University of the Basque Country (UPV/EHU), Paseo de la Universidad 7, 01006 Vitoria-Gasteiz, Spain; albertesponanoguera@gmail.com (A.E.-N.); jeciriza@gmail.com (J.C.); albertocanibano@gmail.com (A.C.-H.); rosa.hernandez@ehu.eus (R.M.H.); 2 Biomedical Research Networking Center in Bioengineering, Biomaterials and Nanomedicine (CIBER-BBN), 01006 Vitoria-Gasteiz, Spain; 3 University Institute for Regenerative Medicine and Oral Implantology - UIRMI (UPV/EHU-Fundación Eduardo Anitua), 01006 Vitoria, Spain; 4 Singapore Eye Research Institute, The Academia, 20 College Road, Discovery Tower, Singapore 169856, Singapore

**Keywords:** Type 1 diabetes mellitus, hydrogel, nanoencapsulation, microencapsulation, macroencapsulation, bioprinting

## Abstract

Type 1 Diabetes Mellitus (T1DM) is characterized by the autoimmune destruction of β-cells in the pancreatic islets. In this regard, islet transplantation aims for the replacement of the damaged β-cells through minimally invasive surgical procedures, thereby being the most suitable strategy to cure T1DM. Unfortunately, this procedure still has limitations for its widespread clinical application, including the need for long-term immunosuppression, the lack of pancreas donors and the loss of a large percentage of islets after transplantation. To overcome the aforementioned issues, islets can be encapsulated within hydrogel-like biomaterials to diminish the loss of islets, to protect the islets resulting in a reduction or elimination of immunosuppression and to enable the use of other insulin-producing cell sources. This review aims to provide an update on the different hydrogel-based encapsulation strategies of insulin-producing cells, highlighting the advantages and drawbacks for a successful clinical application.

## 1. Introduction

Diabetes mellitus (DM) is currently affecting almost 422 million people worldwide and the global incidence rate is predicted to increase to 552 million by 2030, thereby its increasing prevalence has led to consider such disease as an epidemic of the 21st century [1]. Type 1 diabetes mellitus (T1DM) is seen as an increasing health hazard with an estimated prevalence ranged between 5% and 10% of the total cases of DM worldwide mostly affecting young people [2]. T1DM is characterized by the autoimmune destruction of β-cells in the pancreatic islets that lead to a severe insulin deficiency and, the subsequent elevation of blood glucose levels (hyperglycemia). The high glucose levels in the blood lead to devastating micro- and macrovascular complications in diabetic patients, including retinopathy, cardiovascular disease, nephropathy, and neuropathy [3,4]. 

Currently, the therapy based on daily exogenous insulin administration is the primary treatment in patients with T1DM. Managing T1DM requires high patient compliance with daily insulin injections and blood glucose measurements for the rest of their lives [5]. However, this treatment does not mimic the real-time insulin secretion pattern of the β-cells in the pancreas and, therefore, hindering the stringent control of glucose metabolism [6]. In consequence, any carelessness can result in episodes of hyperglycemia, but also there is an increased risk of acute episodes of low glucose levels in the blood (hypoglycemia) that can lead to cognitive impairment, seizures, and coma [7,8]. 

Alternatively, there are other approaches based on the replacement of the damaged β-cells in T1DM patients. In this regard, the whole pancreas transplant was the first approach that was successfully applied in the clinics [9]. This treatment aims to reestablish normoglycemia by the replenishment of the depleted pancreatic islets reserve, thus avoiding issues related to daily exogenous insulin injections [10]. In 1966, the results of the first clinical pancreas transplant showed immediate blood-glucose levels restoration, but the procedure demonstrated very poor implant survival rates; since after six months, less than 8% of transplanted pancreas survived [11]. Nowadays, according to the International Pancreas Transplant Registry, with the advances made in surgical techniques, immunosuppression treatments, and post-transplant monitoring, approximately 80% of transplanted patients achieve a three years survival rate, defined as insulin independence [9]. However, despite the advantages, whole pancreas transplantation still shows many drawbacks such as the scarcity of pancreas donors, the need for long-term immunosuppression and the elevated risk of surgical complications, such as graft pancreatitis, peritonitis, and graft thrombosis, among others [12]. The high risk of morbidity caused by surgical complications and the strong immunosuppressive regimen forced to look for other alternative β-cell replacement methods. 

Interestingly, the mass of pancreatic islets within the pancreas is really low, actually, they only represent 2% of the whole pancreas. Thereby, the transplantation of isolated pancreatic islets appears to be a great alternative for blood glucose levels restoration in Type 1 diabetic patients, as it avoids complications associated with daily insulin administrations and reduces the surgical risks associated with whole pancreas transplantation [13]. The first successful results of islets transplantation were described in 2000, when a research group at University of Alberta (Edmonton, Alberta, Canada) transplanted 800,000 pancreatic islets into the hepatic portal vein in seven patients under a steroid-free immunosuppressive treatment, thus achieving insulin independence for an average of one year in seven patients [14]. This procedure is universally known as the “Edmonton protocol” and, it represented a turning point in the field, provoking a considerable increase in islet transplantation research [15]. Currently, after advances in the islet isolation techniques and in the immunosuppressive treatments, the transplantation of pancreatic islets represents the best approach for T1DM cure, ensuring tight control over glucose metabolism and improving the quality of life of diabetic patients without side effects [14,15]. However, despite the great results, less than 20% of the transplanted patients remain insulin-independent after five years because the Edmonton protocol still displays limitations that hamper the widespread clinical application [16]. In this sense, the most relevant obstacles include the scarcity of pancreas donors, the low islet extraction yield from the whole pancreas and, the loss of a large percentage of islets after the intraportal islet infusion (>60%) [17,18]. Additionally, the survival of the grafted islets is jeopardized due to the poor vascularization at the implantation site that supposes low nutrients supply and hypoxia during the first period after transplantation, thus leading to a potential graft failure [13,14]. Therefore, until these deficiencies are overcome, islet transplantation will remain as a treatment available only for carefully selected cases of severe T1DM. 

In this regard, recent approaches seek to mitigate these issues by means of cell encapsulation techniques [19]. This technology aims to encapsulate therapeutic cells, such as pancreatic islets, within biocompatible materials, with the objective of providing a support structure to the islets that replicates the native islet micro- and macro-environment and offers immunoisolation once implanted [16,19]. Therefore, encapsulation of pancreatic islets prior to transplantation could potentially address some of these issues: (i) overcoming the lack of human donors, as it may allow for transplanting xenogeneic islets and other insulin-producing cell phenotype; (ii) providing a delimited structured scaffold that prevents the islets loss after implantation; and (iii) eliminating the need for long-term immunosuppression [16,19]. Among all the different types of biocompatible materials, the ones with the ability to form hydrogels have received significant attention for pancreatic islet encapsulation [20]. 

**Figure 1:**
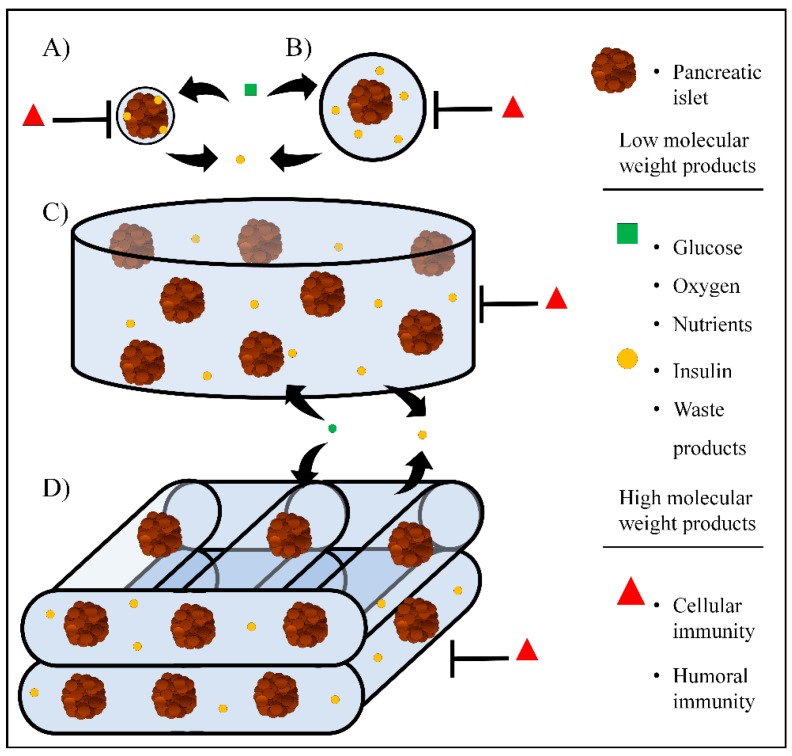
Current hydrogel-based approaches for pancreatic islet encapsulation. (**A**) nanoencapsulation, (**B**) microencapsulation, (**C**) macroencapsulation, and (**D**) 3D bioprinting.

Hydrogels consist of a three-dimensional (3D) network of cross-linked macromolecules and water filling the macromolecular 3D structure [21]. Hydrogel-like biomaterials have demonstrated to be good candidates for pancreatic islet encapsulation because of their good biocompatibility, high-water content, structural and mechanical similarities with the native pancreatic extracellular matrix (ECM) and their permselectivity to low and high molecular weight components, which provides protection against immune cells and high molecular weight immune molecules. In addition, this permselectivity allows the active diffusion of nutrients, oxygen, and therapeutic molecules such as insulin [22]. As a consequence of having a better understanding of the physiological requirements for encapsulation of pancreatic islets and other insulin-producing cell (IPC) phenotypes, new approaches and strategies are constantly being developed into the field. These can be classified into four categories: (a) nanoencapsulation, by placing thin hydrogels films around individual islets (Figure 1A); (b) microencapsulation of small groups of islets, individual islets, or other IPC within spherically shaped hydrogel microcapsules (Figure 1B); (c) macroencapsulation of islets or other IPC within bulk hydrogels that can be shaped and molded within encapsulating devices (Figure 1C); and (d) 3D bioprinted hydrogel-like scaffolds with embedded islets or other insulin-producing cells (Figure 1D) [22,23,24,25].

In this regard, this review aims to provide an update on the pancreatic islet encapsulation within the different aforementioned hydrogel-based approaches for T1DM treatment. Our basic content in this article will be the currently available islet and β-cells encapsulation strategies, analyzing the variables of each technology that require optimization before large-scale application, and highlighting the advantages and drawbacks for a successful clinical application. 

## 2. Nanoencapsulation

Nanoencapsulation is a technique where thin films of a hydrogel are placed onto the surface of a cell aggregate, such as the pancreatic islet, by interfacial polymerization [26]. The final cross-linked hydrogel film results in a nanometric conformal coating placed around the surface of each individual islet or cell aggregate [27,28]. This type of islet encapsulation can be achieved using conformal coatings.

### 2.1. Conformal Coating

**Figure 2:**
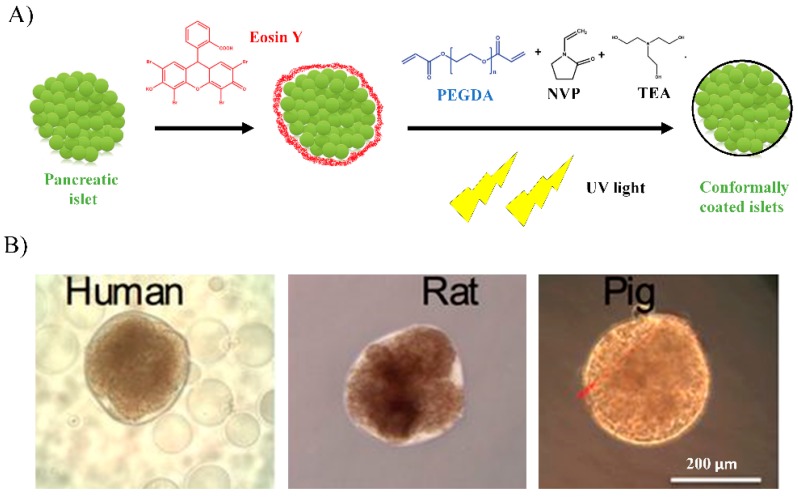
(**A**) Schematic of pancreatic islet nanoencapsulation with polyethylene glycol diacrylate (PEGDA), eosin Y, triethanolamine (TEA), N-vinylpyrrolidone (NPV) by UV light-mediated interfacial photopolymerization. (**B**) Brightfield images of conformally-coated human, rat, and pig islets. Scale bar: 200 μm. Adapted from [32] with permission from Tomei AA et al, Proceedings of the National Academy of Sciences of the United States of America; published by PNAS, 2014.

The most common hydrogel-based technique for conformal coating of the islets is carried out by light-mediated interfacial polymerization of acrylate-based polymers, being the most used biomaterial the acrylated polyethylene glycol (PEG) [29]. The nanoencapsulation of pancreatic islets using acrylated PEG was developed at the University of Texas, patented by Neocrin Inc. and posteriorly transferred to Novocell Inc. [30]. Briefly, for the islets nanoencapsulation, the photoinitiator eosin Y is attached to the islets surfaces. Next, a mixture of acrylated PEG and n-vinylpyrrolidone (NVP) monomers and polymerizing accelerant triethanolamine (TEA) are added to the islets and, finally, islets are exposed to UV light thus cross-linking the acrylates and NVP to form a hydrogel thin film binding to the eosin Y previously bonded at the islet surface (Figure 2A) [31]. 

### 2.2. In Vivo Approaches 

The first in vivo study with nanoencapsulated islets was carried out by Neocrin Inc., where they explored the feasibility of islet xenotransplantation of PEG nanoencapsulated porcine islets in diabetic rat models [33]. Results showed that 5000–8000 nanoencapsulated porcine islets decreased blood glucose levels within the normoglycemic range (<200 mg/dL) when implanted into diabetic rats. However, animals returned to hyperglycemia 60–70 days after implantation [33]. When this approach was translated into non-human primates, nanoencapsulated islets did not succeed in restoring normoglycemia [34]. The islets viability was compromised due to the cytotoxic effects of the co-initiator TEA exposure, required in the polymerization step [35] and, importantly, due to the aggressive immune reaction from animals to the PEG coatings and the xenotransplanted islets that were incompletely coated leaving them partially exposed [36]. 

For these reasons, the islet nanoencapsulation technology was improved to obtain completely coated encapsulated islets with better biocompatibility and higher immunoprotection. In this regard, the company Novocell, Inc. in the attempt to improve the PEG-coating formulations modified the PEG component to enhance binding to photo-initiators and to accelerate the cross-linking reaction in order to be less immunoreactive in large animals and achieve more uniform and full coatings. The company carried out a pre-clinical non-human primate study by implanting nanoencapsulated allogeneic islets into the subcutaneous tissue of the abdomen in five diabetic baboons. Results demonstrated allograft function with complete insulin independence up to 20 months in three of the implanted recipients. The other two implanted animals in this group of five did not achieve insulin independence [30].

### 2.3. Clinical Trials 

Encouraged by the successful results in non-human primates, Novocell launched a phase I/II clinical trial in the USA. The study was approved by the FDA for 12 patients, but only two patients of 25–30 years were approved by Novocell following the inclusion and exclusion criteria to start the study [37]. The implant procedure involved nanoencapsulated allogeneic islets were subcutaneously injected into the back and the abdomen, without the use of long-term immunosuppression. Although the recipients experienced a decrease in the number of hyperglycemic (>300 mg/dL) and hypoglycemic (<70 mg/dL) episodes, none of the patients achieved insulin independence during the first six- and four months post-implantation [30]. For this reason, no more patients were implanted with nanoencapsulated islets and the clinical trial was terminated (Table 1). 

### 2.4. Advantages and Limitations of Nanoencapsulation

In clinical pancreatic islets transplantation programs, the Edmonton protocol is the most standardized approach [14]. However, capsules of higher diameter than the islets themselves may clog narrower blood vessels potentially resulting in severe thrombosis of the liver [45,46,47]. In this sense, nanoencapsulation is the only approach that enables successful transplantation of encapsulated islets through the portal veins following the Edmonton procedure without clogging the portal vein [48].

Another point to take into account is that pancreatic islets are highly variable in size [49]. In this regard, nanoencapsulation technology is the only approach that allows the standardization of the capsule thickness on each islet independently of its size, as the capsule adapts to the shape and size of the islets, resulting in nanocapsules with uniform thickness (Figure 2B) [32,50].

Although nanoencapsulation offers many advantages in comparison with the other encapsulation strategies, this approach has some limitations that difficult its clinical application. The main issue is that, in some cases, islets are exposed because they are not completely coated, which can trigger the host’s immune reaction, resulting into graft failure [36]. Although significant advances have been achieved in the nanoencapsulation procedure to fully coat pancreatic islets, some compounds used for the interfacial hydrogel-film cross-linking such as TEA, as well as the UV light used in the film formation still have a cytotoxic effect on islet viability [35]. Moreover, PEG may infiltrate and interact with the islets causing necrosis [51]. Therefore, further investigation is required in developing less immunoreactive formulations that will not jeopardize the islet survival and satisfy the biological and physical demands of islet grafts [52]. 

From the point of view of the graft biosafety, the nanoencapsulation approach still has limitations which were noted in the only existing clinical trial where recipients did not achieve insulin independence [37]. On the one hand, the ultrathin hydrogel film cannot ensure a high degree of immunoprotection to the transplanted islets due to the uncompleted coating that leads to cell protruding. Therefore, pancreatic islets are not completely hidden from the host immune system, thus potentially leading to graft failure. On the other hand, other potential adverse events might be direct consequences of the difficult removal of the transplanted nanoencapsulated islets, since there is no control over the location of every single nanocapsule [53,54]. For this reason, new approaches must be developed to allow safer nanoencapsulation-based therapies.

## 3. Microencapsulation

**Figure 3:**
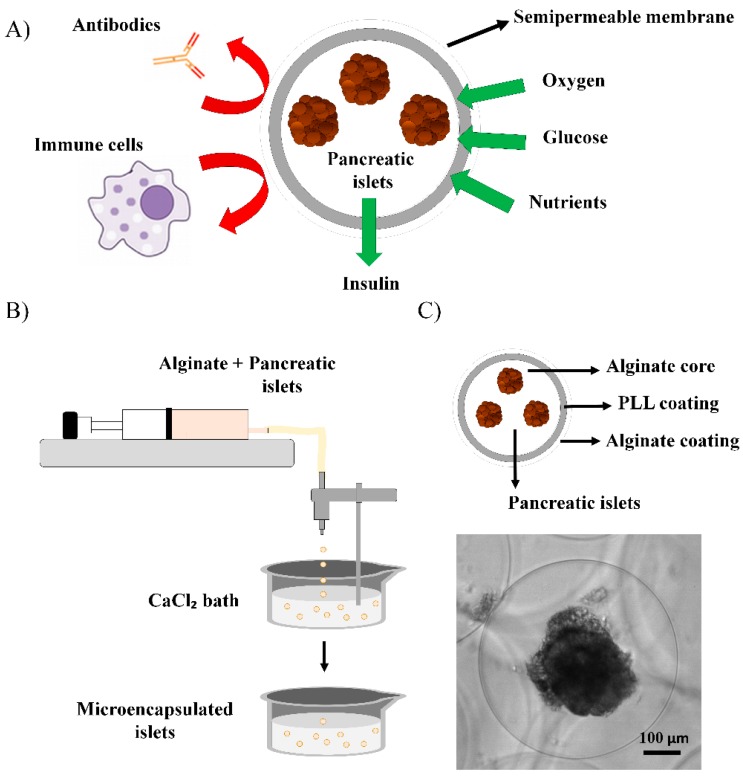
*(***A**) Principle of immunoisolation by a microcapsule. The semipermeable membrane provides protection against macromolecules and cells of the immune system while allowing the diffusion of low molecular weight molecules such as oxygen, glucose, nutrients, and insulin. (**B**) Schematic of pancreatic islet microencapsulation within alginate microcapsules obtained by ionic cross-linking in a CaCl_2_ bath where droplets that are generated by an electrostatic droplet generator are gelled. (**C**) Layers structure of an alginate-poly-L-lysine-alginate (APA) microcapsule and, brightfield image of a microencapsulated pseudoislet generated from the rat insulinoma INS1E cell line. Scale bar: 100 μm.

Microencapsulation technology consists of embedding single-cells or microtissues a hydrogel like polymer in a spherical shape (Figure 3A) [55,56]. The major challenge that affects the clinical applicability of the microencapsulation technology for pancreatic islet transplantation is the biocompatibility of the encapsulation material [57]. Biocompatibility determines the performance of microcapsules for allowing the long-term survival of the therapeutic cells [58]. A wide range of biocompatible polymers have been used in microencapsulation applications such as collagen, cellulose, agarose, chitosan, gelatin, PEG, poly-methyl methacrylates and 2-hydroxyethylmethacrylate; but with low performance in islet encapsulation [23,59]. Among all, alginate is the most used biomaterial, as it displays high biocompatibility for both the cells in the microenvironment at the implantation site and for the islets inside the microcapsules [60].

This biomaterial is a natural anionic polymer extracted from brown seaweeds that consists of linear blocks of (1,4)-linked β-*D*-mannuronate (M) and α-L-guluronate (G) residues. The most common method to prepare alginate hydrogels as microcapsules is the ionic cross-linking [61]. Briefly, the alginate solution containing islets is dropped through a nozzle into a solution with divalent cations such as calcium or barium ions, where microcapsules of 250–1000 µm in diameter are finally gelled, thereby entrapping one or more pancreatic islets (Figure 3B) [62]. The great biocompatibility and the easy formation process of microcapsules under mild conditions makes this biomaterial an excellent candidate for pancreatic islet microencapsulation, that has been already successfully applied in in vivo studies and clinical trials [63].

### 3.1. In Vivo Approaches 

The use of alginate-based microcapsules containing pancreatic islets in the T1DM treatment has shown great promise since many studies have shown the successful restoration of the blood glucose levels in diabetic animal models [64]. 

In 1980, Lim and Sun described for the first time success in reversing diabetes disease by transplantation of encapsulated islets within alginate microcapsules [63]. Since then, alginate microencapsulation gained progressive popularity, and research focused on improving microencapsulation material formulations to achieve appropriate immunoprotection, biocompatibility, and mechanical stability for pancreatic islet transplant purposes. 

In this regard, many studies aiming diabetes reversal used alginate microcapsules for islet microencapsulation coated by a polycation thin layer, such as polyallylamine, poly-*L*-lysine (PLL), poly-L-ornithine (PLO), or polyvinylamine to obtain better control of diffusion and permselectivity, as well as to improve the mechanical stability of the microcapsule system [30]. However, some of those polycations display cytotoxic and proinflammatory effects. For example, PLL coatings on microcapsules at higher concentrations than 0.05% are toxic to several cell types such as β-cells, T-lymphocytes and monocytes. Additionally, PLL may enhance the fibrotic and inflammatory responses against the microcapsules when implanted into Balb/c mice through the induction of pro-inflammatory cytokines such as the Tumor Necrosis Factor (TNF) [65]. For this reason, another external alginate coating is often added to the microcapsules to hide the polycation layer. Thereby, a typical double-coated alginate microcapsule by a semipermeable polycationic layer and an alginate outer shell displays an improved biocompatibility, while maintaining the advantages of the polycation coating (Figure 3C) [66,67]. Several in vivo studies in diabetic *Cynomolgus* monkeys have been carried out with alginate–poly-*L*-lysine–alginate (APA) microcapsules, which reduced the blood-glucose levels to healthy values with insulin independence [68]. Despite these encouraging results, the main factor that seemed to lead to graft failure with APA microcapsules was islet necrosis caused by high fibrotic response against the microcapsules, low oxygenation and the PLL degradation over time [69]. 

To overcome the problems associated with PLL in APA microcapsules, Elliott et al. modified the microcapsule composition with a PLO coating instead of PLL. These microcapsules containing islet allowed to restore normoglycemia in diabetic *Cynomolgus* primates, while improving the islet survival [70]. Other authors directly used polycation coating-free microcapsules, to avoid the potential cytotoxic and pro-inflammatory effects of polycations, by cross-linking the alginate microcapsules with barium ions [54]. These barium-cross-linked microcapsules have shown higher strength and less permeability to high molecular weight components of the immune system such as IgG in comparison with microcapsules cross-linked with calcium [71]. Thereby, the absence of polycations in the microcapsules formulation makes these barium-cross-linked alginate microcapsules more biocompatible compared to APA microcapsules [72]. However, even in the absence of polycations, the barium alginate microcapsules still are susceptible to fibrotic overgrowth [54].

Overall, the fibrotic and inflammatory response against the microencapsulated islets, or the microcapsule material itself, mostly determine the success of the grafted microcapsules. However, this reaction is still poorly understood. In fact, in addition to the use of polycations in the microcapsules formulation, many other factors can be involved in the immune response such as the alginate purity, the biocompatibility of the materials conforming the microcapsule, the alginate chemical composition (guluronic/mannuronic ratio) and the size of the microcapsules [24,73,74]. 

### 3.2. Clinical Trials 

In 1994 Soon-Shiong et al., performed the first successful clinical trial with APA microencapsulated allogeneic islets transplanted into the peritoneum of a diabetic patient under immunosuppression treatment (Table 1) [38]. Results demonstrated that the grafted microcapsules allowed to restore control over glucose metabolism with insulin independence for nine months. Following this initial trial, Calafiore et al., transplanted intraperitoneally alginate-PLO microencapsulated human islets without immunosuppression. Several weeks after transplantation, patients decreased the exogenous insulin requirements without side effects derived from the implantation procedure. Moreover, there was no evidence of immune sensitization from the hosts (Table 1) [39]. Later, Tuch et al., transplanted four diabetic patients into the peritoneal cavity with allogeneic islets in barium-cross-linked microcapsules without the use of immunosuppression, resulting in a decrease in blood glucose levels, but not enough to reduce the insulin requirements (Table 1) [40]. 

Although initial clinical trials mainly focused on the use of human pancreatic islets, the permselective immunobarrier of microcapsules allows the safe and efficacious use of xenogeneic islets for transplantation, thereby offering an alternative source of cells with the ability to secrete insulin that could help to overcome the shortage of pancreas donors. In this regard, transplantation of microencapsulated porcine islets commenced in Type 1 diabetic patients [37]. In 2007, the Living Cell Technologies (LCT) Company performed a larger clinical study using commercial pig islets within alginate-PLO microcapsules which were called “Diabecell”, where eight patients received varying islet doses (5000–10,000 IEQ/kg body weight) (Table 1). Six patients displayed a reduced exogenous insulin requirement for up to eight months, thereby demonstrating the potential use of this technology as a safe and effective approach for T1DM treatment [41]. 

### 3.3. Advantages and Limitations of Microencapsulation

In most tissues, it has been shown that maximum diffusion distance for effective oxygen and nutrient diffusion from blood capillary to cells is about 200 μm [75]. Higher diffusion distances induce a gradient of nutrients and oxygen from the cell encapsulation system surface to the center of the cells, which may affect cell function and survival [75]. In this sense, the reduced diameter and the large surface area-to-volume ratio of microcapsules results in improved diffusion, thereby making this encapsulation system preferable over others, such as macroencapsulation, where longer diffusion distances hardly compromise oxygen and nutrients diffusion [23]. This fact also has a direct impact on the graft function as the response of microencapsulated islets to glucose changes in the bloodstream is faster and more effective than in the macroencapsulation systems [24]. 

Another advantage of microencapsulation is that the survival of the islets is potentially higher than in the macroencapsulation approaches. In the scenario where one microcapsule breaks or it is attacked by the immune system, it will only affect the islet encapsulated in that specific microcapsule while the rest of the microencapsulated islets will be not affected [23]. In contrast, in case of partial graft failure of islets encapsulated in a macroencapsulation device, the entire graft is at risk of destruction by the host immune system, as they are contained in a single encapsulation device [30]. Regarding the immunoprotection, in the mentioned clinical trial with APA microcapsules, patients required immunosuppressive treatments to ensure the graft survival [38]. However, clinical trials using PLO microcapsules did not use immunosuppression and there was no evidence of immune sensitization from the hosts [39]. Therefore, using an appropriate coating, microcapsules may represent a promising approach for safe and effective therapy for T1DM treatment without immunosuppression. From another point of view, another advantage is that, microcapsules can be implanted using a minimally invasive procedure thanks to their reduced size. Additionally, the smooth spherical shape of the microcapsules minimizes the foreign body reaction as opposed to host inflammatory reactions detected against rough surfaces [54]. In contrast, the major limitation to translate the microencapsulated islet to the clinics is the lack of large-scale microcapsules production systems. Currently available methodologies for cell microencapsulation are not able to efficiently encapsulate large numbers of islets in a reasonable amount of time, which may result in hypoxic stress that can cause the loss of islet function and reduce the viability [76]. Moreover, this technology has another technical obstacle that relies on the large therapeutic graft volume that may enhance the host immune reaction after implantation; this occurs due to two factors: the very small volume of islets compared with the large volume of the encapsulating hydrogel-like material, and the too elevated number of empty microcapsules without islets that are produced during microencapsulation process [77,78]. In this regard, aiming to reduce the microcapsules graft volume, separation of microencapsulated islets from non-therapeutic microcapsules is usually accomplished by hand selection; however, the separation procedure is slow and tedious, thus complicating its reproducibility [77,78]. Recently, a novel microcapsule sorting system allows for the separation of magnetically labeled microencapsulated islets from empty microcapsules [64]. This purification system is based on a magnetic separation through a microfluidic device containing magnets, which guide the magnetized microencapsulated islets towards an output channel, while the empty microcapsules are eliminated through a different output channel. This technology allows high purification yields while avoiding manual steps thereby minimizing technical errors and improving the reproducibility of the separation process. Overall, the too high graft volume and the large microcapsules diameter impede the transplantation of microencapsulated islets into the liver following the standard Edmonton procedure, as it would suppose the high risk of thrombosis. Alternatively, microcapsules containing islets have been transplanted into the peritoneal cavity in most clinical trials, where large volume of microcapsules can be implanted without clogging the vascular system [39,40,41]. However, the indirect access to blood within the peritoneum leads to lower availability of nutrients and oxygen, which can compromise the islet survival, thereby reducing the probability of success [79]. In addition, with the use of microencapsulated islets, as it happens with nanoencapsulated islets, there is no control over the location of every microencapsulated islet and, subsequently, microcapsules are difficult, if not impossible, to retrieve completely after implantation, thereby affecting negatively to the biosafety of this approach [40,80,81].

## 4. Macroencapsulation

Hydrogel-based macroencapsulation systems are macroscopic encapsulation devices (>1 mm) containing a large number of pancreatic islets or insulin-producing cells, thereby allowing the delivery of a curative β-cell dose within only one or very few devices [23]. The described hydrogel-based macroencapsulation systems to date are extravascular devices. In this sense, the major drawback of extravascular approaches is the limited oxygen diffusion and nutrient transport at the implantation site, which tends to result in a reduction of cell viability and loss of function [44,82]. Research on macroencapsulation systems focuses on the development of strategies and device configurations that can provide sufficient oxygen and nutrients to transplanted islets or insulin-producing cells.

### 4.1. In Vivo Approaches

Macroencapsulation systems involving hydrogels are mostly circular or planar devices consisting of islets embedded in hydrogels placed within a semipermeable chamber. This encapsulation system is designed to prevent the aggregation and clustering of the therapeutic cells inside the device while protecting the cells from mechanical stress after implantation [83,84]. 

Islet sheet is one example of the planar flat sheet devices designed by Islet Sheet Medical, which involved a multilayered construct of alginate composed of a central alginate layer containing islets, and two external acellular alginate layers covering the central layer for immunoprotection. This device has been demonstrated to provide excellent graft survival in diabetic dogs, achieving normoglycemia for 12 weeks [85]. More recently, Dufrane et al. developed a planar flat sheet device, called a monolayer cellular device, consisting of a monolayer of collagen matrix where islets are embedded, and two alginate layers covering both sides of the collagen matrix [86]. Encapsulated porcine islets within this device were implanted subcutaneously and demonstrated to correct hyperglycemia for up to 6 months in diabetic monkeys without immunosuppression. Although a strong immune response was detected after transplantation, a total impermeability of alginate layers to IgG was demonstrated up to 20 weeks [86].

**Figure 4:**
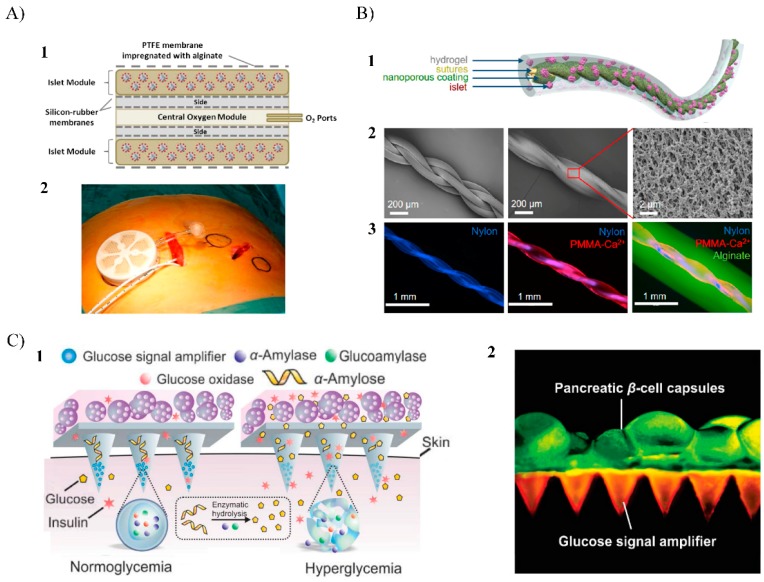
(**A**) β-Air macroencapsulation system for islet encapsulation: (1) Schematic view of the chamber system. The core of the device has a gas module, connected to access ports for exogenous oxygen refilling. Separated by gas permeable membranes, two compartments surround the gas module that houses pancreatic islets immobilized within alginate; (2) chamber system with integrated human islet graft with connected access ports before implantation. Adapted from [44] with permission from Barbara Ludwig et al, Proceedings of the National Academy of Sciences of the United States of America; published by PNAS, 2013. (**B**) TRAFFIC islet macroencapsulation device: (1) Schematic illustration of the design of TRAFFIC; (2) SEM images of the thread and the uniform nanoporous surface modifications; (3) fluorescent microscopic images of the thread, modified thread, and the complete TRAFFIC device. Adapted from [92] with permission from An D et al, Proceedings of the National Academy of Sciences of the United States of America; published by PNAS, 2018. (**C**) Microneedle patch system with integrated pancreatic cells and synthetic glucose-signal amplifiers: (1) Schematic of the glucose-responsive system functioning of the microneedle patch where glucose signal amplifiers (GSA) promote insulin release triggered by a hyperglycemic state; (2) Fluorescence microscopy image of the microneedle patch: patch was loaded with rhodamine-labeled GSA and calcium AM-stained pancreatic β-cells were positioned on the back of the patch. Scale bar: 500 μm. Adapted from [93] with permission from Ye Y et al., Advanced Materials; published by Wiley Online Library, 2016.

The major disadvantage with the macroencapsulation devices is the poor oxygen diffusion throughout the outer semipermeable membrane compromising the viability of the implanted islets, thereby limiting their ability to secrete insulin and leading to graft failure. This issue was addressed by another macroencapsulation system, an oxygen-refueled device called β-Air, which was developed by Beta-O_2_ Technologies Ltd. [43]. This device consists of a semipermeable chamber containing islets embedded within an alginate hydrogel and, an additional compartment that enables daily oxygen supply through an external tubing system (Figure 4A). Interestingly, the design of the β-Air allows fabricating this device in different sizes. In this sense, small β-Air devices used in animals (external diameter: 31.3 mm, height: 7 mm) can be scaled to larger ones to be used in clinical trials in humans (external diameter: 68 mm, height: 18 mm) [43,87]. Preliminary studies with the small size β-Air devices implanted in diabetic pigs showed that the function of encapsulated allogeneic islets was preserved, and blood glucose levels were decreased to normal values for several months [87,88]. Regarding the oxygen supply strategy, perfluorocarbons and calcium peroxide (CaO_2_) as a source of oxygen has been added within hydrogel formulations containing β-cells in in vitro experiments, having a considerable impact on cells with high oxygen uptake rates and enhanced cell viability and metabolic activity [89,90]. In this sense, the inclusion of this kind of oxygen carriers in the encapsulation matrix could be a useful strategy for overcoming the oxygen limitations, ensuring cell viability and functionality of β-cells within macroencapsulation devices. 

In attempt to address the low oxygen supply from another point of view, the BioHub macroencapsulation system was developed at the Diabetes Research Institute of Miami aiming a high graft vascularization [66]. The novelty of this technology is the use of an injectable biomaterial for islet encapsulation, which consists of a hydrogel-like matrix made with plasma from the own patient and thrombin. This hydrogel degrades over time, leaving the islets intact, while allowing and promoting the formation of new blood vessels that supply oxygen and nutrients to the islets, thereby supporting their survival and function [91]. After achieving good results in small animals, further studies were performed in diabetic *Cynomolgus* monkeys. On this matter, allogeneic islets were injected using BioHub system in the omentum of diabetic monkeys under immunosuppressive treatment. A few weeks after implantation, recipients showed progressive reduction of exogenous insulin requirements. After retrieving the grafts 49 days post-implantation, histopathologic analysis demonstrated well-preserved islet morphology with abundant internal and external vascularization [91]. 

Recently, other macroencapsulation devices geometries have been developed to enhance the diffusion properties and, to improve their retrievability and replaceability. For example, the thread-reinforced alginate fiber for islets encapsulation (TRAFFIC) device, which involves an alginate hydrogel layer with controllable thickness containing islets placed around a nanoporous calcium-releasing central nylon thread (Figure 4B) [92]. The device can be extended to meters long allowing to scale-up the system to large animals and, still be entirely retrievable through minimally invasive surgery. In vivo studies in mice showed that TRAFFIC encapsulating rat islets restored normoglycemia in diabetic mice while providing immune protection for up to three months. Additionally, no significant fibrotic overgrowth was noted on the device. Similarly, the TRAFFIC device scalability and retrievability was also demonstrated in dogs [92].

Other authors have developed another novel β-cell encapsulation device that is placed externally onto the skin, thereby eliminating the immune response caused in more invasive implantation procedures and avoiding the use of immunosuppression [93]. This system involves a microneedle patch composed of hyaluronic acid hydrogel housing insulin-producing cells and glucose signal amplifying enzymes (GSA) (Figure 4C). With this device, in a hyperglycemic state, glucose diffuses inside the microneedles and, next, the glucose signal is amplified through GSA thus stimulating insulin secretion from encapsulated β-cells, which is secreted through the microneedles in a minimally invasive manner. In in vivo studies with diabetic mice, a microneedle patch responded rapidly to hyperglycemia leading to the stabilization of blood glucose levels for 10 hours [93]. This macroencapsulation device displayed a promising alternative to pancreatic β-cells internal implantation for glucose homeostasis regulation, but further work is required for improving the glucose diffusion and the viability of encapsulated cells.

### 4.2. Clinical Trials

There have been only few clinical trials testing macroencapsulated islet using some of the abovementioned macroencapsulated islet or β-cells products. In addition, only the Beta-O_2_ company has published clinical results with the β-Air device, while other researchers evaluating other macroencapsulation devices had made oral presentations at public meetings. 

For example, in 2010 Dufrane et al., encouraged by the results obtained in the preclinical studies in diabetic monkeys [86], implanted subcutaneously the Monolayer Cellular Device containing allogeneic islets in a 74-year-old type 1 diabetic patient (Table 1) [42]. Blood glucose levels were controlled for 361 days after transplantation, along with a notable reduction of hypoglycemic episodes. Implant retrieval after a year revealed the macroscopic integrity of the device without signs of inflammation and immunization against the donor cells. No more details of this clinical trial have been published. 

Later, in 2012, a pilot clinical trial was carried out with allogeneic islets (2100 IEQ/kg body weight) encapsulated in the β-Air device that was implanted in a 63-years old patient. In this study, graft function was achieved for 10 months with controlled blood glucose and insulin secretion regulation, while preserving the islet morphology and function without immunosuppression (Table 1) [43]. Encouraged by the good results in terms of glucose metabolism control and safety signals without serious adverse effects of β-Air device implantation, another clinical trial has been conducted to continue evaluating the safety and efficacy of this system into type 1 diabetic human patients [94]. In this study, four patients were implanted with 1800–4600 islet equivalents per kg/ body weight and were monitored for 3-6 months. Results reinforced the preliminary results where implantation of the β-Air device was safe and successfully prevented immunization and rejection of the transplanted islets. However, although this device can support survival of allogeneic islets for several months, it cannot achieve long-term insulin independence due to limited function of transplanted islets. 

More recently, the mentioned BioHub macroencapsulation system has been evaluated in an ongoing clinical trial, with the objective to demonstrate the safety and long-term feasibility of this approach (Table 1) [44]. In such study, a 43-year-old diabetic woman was implanted in the omentum with approximately a total dose of 600,000 allogeneic IEQ encapsulated within the BioHub system [95]. This approach demonstrated promising results, as the patient experienced a restoration of glucose homeostasis with insulin independence for 12 months. 

### 4.3. Advantages and Limitations

The main advantages of macroencapsulation technology over nano- and microencapsulation is the easy implantation of the islets in a precise location of the body in one single device and the possibility to simply retrieve the device in case of graft failure or other complications, as it was demonstrated with the mentioned TRAFFIC and microneedle patch devices [92,93]. From another point of view, although there are other types of macroencapsulation devices without involving hydrogels that can also hold high amounts of islets [82], in most cases islets are freely floating inside the encapsulation chamber, resulting in aggregation of islets that negatively affects islet structure, limits the nutrients and oxygen diffusion, and leads to loss of islet function, apoptosis, and death [79]. In contrast, macroencapsulation devices embedding islets within hydrogels provide physical separation of the islets preventing aggregation, maintaining the islet’s rounded morphology, improving the oxygen and nutrients supply to all encapsulated islets thereby enhancing their viability and survival [83,84]. However, some hydrogels are fragile and not stable enough to support the transplanted islets over a long period and, therefore, the long-term islet survival cannot be guaranteed [6]. For this reason, introducing hydrogel-islets biosystems within macroencapsulation devices, like in the β-Air device, has become a promising strategy that confines hydrogel-islets structures, conferring strong mechanical protection that results in improved integrity of the inner hydrogel [43,95]. In addition, in the mentioned clinical trial using the β-Air device [94], results showed that such device prevented immunization and rejection of the transplanted islets. Therefore, it demonstrated that macroencapsulation devices can display a high degree of immunoprotection for safe islet transplantation therapies. However, in the case of the BioHub macroencapsulation system, immunosuppression was required [96]. In this sense, the encapsulating biomaterial and the specific design of the macroencapsulation system define the degree of immunoprotection, which affects directly to the graft survival. Therefore, with an adequate strategy, macroencapsulation devices could be successfully used for islet transplantation without immunosuppressive treatments.

Although interesting benefits come from using macroencapsulation approaches, additional issues must be addressed. As islets exhibit elevated oxygen consumption rates compared to other cell types [97,98], the primary limitation of the macroencapsulation devices compared to nano- and microencapsulation is the reduced oxygen supply due to larger diffusional distances between the encapsulated islets and the outside of the macroencapsulation device [44]. These types of macroscopic devices are usually implanted in the subcutaneous space, which presents low vascularization that affects the islet viability, and subsequently the glycemic control [99]. In this regard, different strategies to address this limitation include oxygen-perfused approaches such as β-Air device [43], bioactive hydrogels like BioHub that promotes vascularization [44], and/or the infusion of vasculogenic factors such as the Vascular Endothelial Growth Factor (VEGF) to stimulate greater vascularization at the device surface [100,101]. However, further investigation is required for adequate oxygenation to achieve long-term islet survival.

Additional issues limit the success of macroencapsulation devices such as cellular adherence and fibrotic response to the outer surface of the device. In this regard, the way that an implantable device interacts with the host’s biological environment and immune system at the implantation site determines the success of the graft [102]. This fact depends on the device size, geometry, and configuration and most importantly, depends on the physicochemical properties of the surface of the device, such as chemical composition, degree of hydrophilicity, roughness, morphology, and pore size. However, it is still not well known how they influence the inflammatory and fibrotic response [103]. In this sense, it is essential to explore macrodevice designs and surface properties to achieve a successful and functional graft during the short and long-term after implantation.

## 5. Bioprinting

Bioprinting is an innovative approach based on the automated additive manufacturing, which offers the potential to assemble tissue-like structures by the controlled positioning of cells, biomaterials, and cell-laden biomaterials individually or in tandem, by stacking layer-by-layer at the desired location [104,105]. In addition, biologically active components can also be added and precisely patterned, such as proteins, genes, and drugs, to better guide tissue generation and/or formation [106]. Thereby, bioprinting technology has great potential to address the increasing demand for organ transplants, thus being a key technology to step forward in the field of regenerative medicine for tissue and organ repair or replacement [107,108]. 

Hydrogel-based biomaterials used in bioprinting are called bioinks and can be loaded with cells or any biological component [109,110,111]. The possibility for multi-bioink fabrication may allow the creation of pancreatic tissue-like structures, where pancreatic islets and other cells present in the pancreas could be included and positioned in the tissue construct similarly to physiological conditions [112]. Currently, β-cell and islet bioprinting has been studied using two different bioprinting systems, inkjet- and extrusion-based printing techniques.

### 5.1. Bioprinting Technology Fundaments 

#### 5.1.1. Inkjet Bioprinting

**Figure 5:**
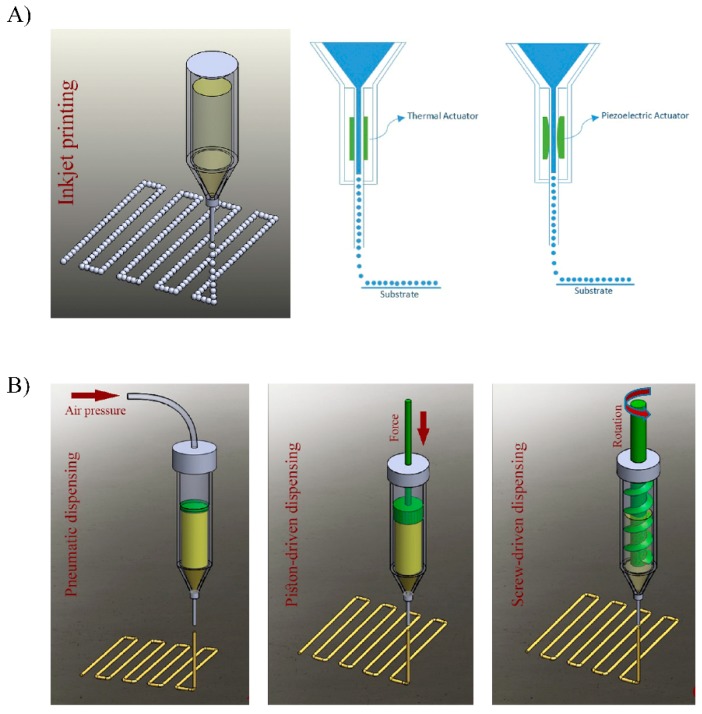
(**A**) Diagram of the inkjet-based printing method using thermal and piezoelectric actuators. (**B**) Diagram of common extrusion-based bioprinting methods: pneumatic, piston-driven, and screw-driven dispensing methods. Adapted from [113] with permission from Derakhshanfar S. et al., Bioactive Materials; published by KeAi, 2018.5.1.2. Extrusion Bioprinting.

Inkjet-based bioprinting utilizes piezoelectric- or thermal-driven mechanisms to dispose picoliter-sized droplets of bioinks with high resolution [113]. Such bioinks are deposited through a nozzle or multi-nozzle system in a high-throughput manner with positional accuracy on the microscale [107]. Piezoelectric and thermal inkjet printing are shown schematically in Figure 5A. This printing process enables fast fabrication speeds maintaining high cell viabilities, however, this approach is restricted to low viscosity bioinks (<10 mPa/s), thereby limiting its applicability [114,115]. In addition, in some cases, cell densities applicable in this technology may be lower than physiologically relevant numbers (<10^6^ cells/mL) due to the increased possibility of nozzle obstruction problems [113]. Another limitation of this approach is that it uses bioinks that possess relatively weak mechanical properties, thus limiting the printing of structures requiring higher mechanical properties [116]. For all these reasons, inkjet printing as a 3D bioprinting technology is still in the early stage of development and its applications are limited compared to extrusion-based studies.

Currently, most of the commercially available bioprinters are extrusion-based, with bioinks driven through a single or multiple nozzles by a pneumatic (air pressure or mechanical screw/piston-driven) dispensing system (Figure 5B) [113,117]. In this approach bioinks are spatially disposed under computer-controlled motion, resulting in the precise deposition of cells encapsulated within the bioink as micrometric cylindrical filaments allowing the desired 3D custom-shaped structures [117]. This rapid fabrication technique provides better structural integrity compared to inkjet bioprinted constructs due to the continuous deposition of filaments. In addition, the extrusion-based technique enables the use of bioinks with a wider range of viscosity (from 0.1 to 30 × 10^7^ mPa/s), incorporating higher working cell densities and/or other biological compounds, and even the incorporation of larger cellular structures such as cell pellets, tissue spheroids, and tissue strands [114,118]. Further comparison with inkjet bioprinting, although the extrusion-based bioprinting technique displays great printing speed, this fabrication speed is lower than in inkjet printing. In addition, the printing resolution is also lower (>200 µm). Additionally, depending on the size of the nozzle diameter and pressure of extrusion, cell viability values may be lower than that obtained with inkjet printers (40%–80%) [116,119]. 

Overall, extrusion-based bioprinting can be considered as the most promising 3D printing approach, as it allows producing organized multicomponent constructs of clinically relevant sizes within a feasible fabrication time.

#### 5.1.2. Bioprinting Process Stages

**Figure 6:**
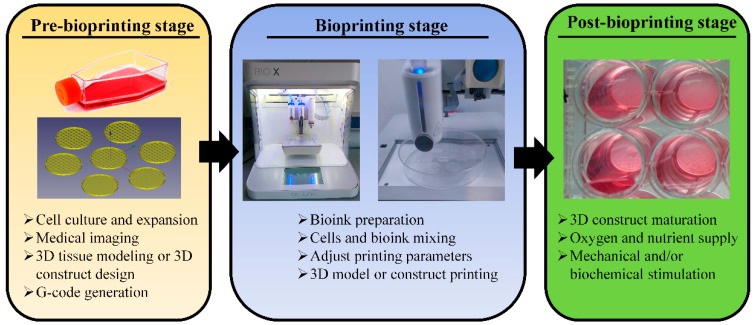
Stages in the 3D bioprinting process: pre-bioprinting, bioprinting, and post-bioprinting with their most important steps.

The complete bioprinting process to generate tissue constructs principally involves the three key stages from the 3D design phase to post-bioprinting steps: pre-bioprinting, bioprinting, and post-bioprinting (Figure 6) [120].

In the pre-bioprinting step, the 3D construct designs can be generated using 3D design software or, they can be obtained from biomedical diagnostic image acquisition techniques directly from patient’s anatomy, such as 3D laser scanning, micro-computed tomography (μ-CT), and magnetic resonance imaging (MRI) [116,121]. After obtaining the 3D construct design, such a design must be converted to G-code, the language consisting of commands that have an assigned movement or action that guide the bioprinter to fabricate the 3D structure layer-by-layer [122,123]. Once the G-Code is obtained, the bioprinting stage begins, which involves three elements: the bioink, the bioprinter, and the bioprinting process [120].

First, the bioink(s) used for the tissue construct printing must be defined and characterized for adequate printability in the different bioprinting modalities [124]. In this regard, the bioink viscosity is the most important factor for successful 3D structures fabrication [108]. Importantly, depending on the bioprinting modality (inkjet- or extrusion-based modalities), bioinks must possess different viscosity requirements [125]. Bioinks used in inkjet bioprinting must have low viscosity so that they can easily flow through the printing nozzle without obstruction issues [116]. For appropriate inkjet bioprinting, bioinks must display a rheopectic behavior, a dilatant behavior that enables the formation of the droplets during the bioink ejection [126]. Moreover, an important post-printing characteristic is that the bioink should immediately solidify after deposition to allow the 3D structure formation [116]. On the one hand, bioinks in extrusion-based bioprinting must show thixotropic shear thinning behavior [113]. Such bioinks exhibit low viscosity when shear forces are applied during the extrusion process. However, bioinks present high viscosity at rest after the extrusion process, thus allowing the formation of 3D structures [119]. In this sense, bioinks can be extruded at low shear forces, thereby protecting cells from physical stress and, then, reform to achieve high fidelity printed structures [105,119,126]. 

Once the adequate bioink is selected, tissue-specific cells that will be included within the bioink must be isolated and/or differentiated and expanded in culture before mixing with the bioink [116]. After obtaining the cell-laden bioink, the working pressure and the printing speed to achieve great printing resolution, which is mainly defined by the bioprinter characteristics and bioink printability [127,128,129]. Finally, the bioprinted tissue structure is required to become mature in suitable bioreactors. This step occurs in the post-bioprinting stage were tissue-like constructs are cultured under controlled conditions, where cells are biochemically and/or mechanically stimulated to promote cell–cell and cell–bioink matrix interactions, thereby achieving the desired biological tissue-like characteristics [120]. 

### 5.2. Early Approaches

Nowadays, the development of a tissue-like pancreas containing pancreatic islets using 3D bioprinting is still an early stage and, limited in vitro and in vivo work has been performed to date. 

**Figure 7:**
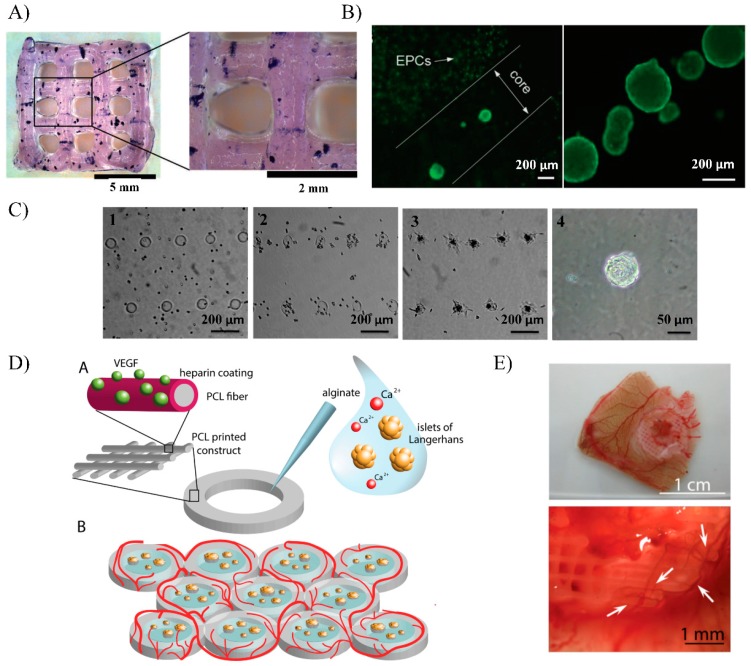
(**A**) Bioprinted scaffold by extrusion-based printing with a bioink composed of alginate and methylcellulose where islets are stained for metabolic activity with MTT one day after bioprinting. Scale bars: 5 mm and 2 mm. Adapted from [130] with permission from Duin S. et al., Advanced Healthcare Materials; published by Wiley Online Library, 2019. (**B**) A coaxial printed construct with encapsulated islets in the core and endothelial progenitor cells (EPCs) in the outer shell. Scale bar: 200 μm. Adapted from [131] with permission from Luis X. et al., Advanced Healthcare Materials; published by Wiley Online Library, 2019. (**C**) Microscopic images illustrating the formation of pancreatic progenitor cell (PPC) clusters on anisotropic tobacco mosaic virus (TMV) particles conjugated with RGD (arginine−glycine−aspartate) patterned spots at 0 h (1), 3 h (2), 24 h (3), and three days (4) after seeding. The scale bars of 1−3 and 4 are 200 μm and 50 μm, respectively. Adapted from [132] with permission from Yang J et al., ACS Applied Materials & Interfaces; published by ACS publications, 2015. (**D**) Schematic of the hybrid polycaprolactone (PCL)/alginate scaffold concept. 3D plotted PCL rings were covalently functionalized with a heparin layer. Heparin was used as an active linker to bind VEGF and protect it from degradation. Islets were encapsulated in the inner part of the structure using alginate hydrogel. Multiple constructs can be printed one next to the other in a honeycomb configuration increasing the available surface for islets embedding and revascularization of the scaffold. Adapted from [133] with permission from Marchioli, G. et al., Advanced Healthcare Materials; published by Wiley Online Library, 2016. (**E**) CAM assay performed with heparin-coated PCL scaffolds loaded with VEGF. Scaffold with 200 ng load VEGF induces blood vessel formation, with normal morphology (Arrows indicate the blood vessel formation). Adapted from [133] with permission from Marchioli, G. et al., Advanced Healthcare Materials; published by Wiley Online Library, 2016.

Duin et al. developed a bioink composed of 3% alginate and 9% methylcellulose to be used for pancreatic islets encapsulation within mesh pattern obtained through extrusion-based bioprinting (Figure 7A) [130]. In this study, pancreatic islets from Wistar rats were successfully embedded within bioprinted constructs without affecting their morphology, while preventing their aggregation. Cell viability and biological function were also evaluated showing viability values around 80% and glucose responsiveness for up to seven days in culture. More recently, researchers from the University of Wollongong in Australia also used an alginate/gelatin bioink for pancreatic islet extrusion-based bioprinting, but with a step forward. They developed a more advanced 3D construct design to enhance islet immunoprotection and promote vascularization [131]. In this work, the printed structure consisted of a multicellular construct accommodating mouse pancreatic islets and endothelial progenitor cells (EPCs). Both cells were successfully disposed forming an inner core containing functional islets that were covered with an outer protecting shell with embedded EPCs (Figure 7B). In this approach, the outer shell seeks to provide immunoprotection to the islets improving immunoisolation, while it simultaneously contains supporting cells, such as EPCs, that can promote the vascularization of the graft. 

Currently, the inkjet-based bioprinting technology is also being developed for patterning β-cell in different construct designs. For example, Yang, et al., used this technology to pattern spots composed of anisotropic tobacco mosaic virus (TMV) particles conjugated with RGD (arginine−glycine−aspartate) that support and control the formation of pancreatic progenitor cell (PPC) clusters [132]. With this approach, the author aimed to establish a novel method to obtain insulin-producing β-cells from PPCs through the expansion and differentiation of 3D printed PPCs size-controlled clusters. Results showed that PPCs were able to adhere onto the multiple printed cell-adhesive spots and form cell aggregates in uniform size and shape, thereby obtaining a robust and reproducible PPCs-cluster patch (Figure 7C). Due to the early stage of inkjet-based bioprinting for diabetes treatment applications, no more studies have been described to date. 

However, some hydrogels are fragile and not stable enough to guarantee long-term islet survival [6]. For this reason, bioprinted constructs with hydrogel-based bioinks can be combined with more stable and stiffer macroencapsulation structures that can be printed simultaneously through another 3D printing modality called fused deposition modelling (FDM) [134]. This modality is based on the extrusion of heated plastic filaments through a nozzle tip to deposit layers onto the printing platform to build 3D objects layer by layer directly from the digital model [135]. The materials used in FDM cannot be mixed with cells, but some of these materials are biocompatible and can be combined with hydrogel-based structures containing cells, thereby obtaining hybrid constructs with enhanced mechanical properties that improve the integrity and stability of the hydrogel cell-laden printed part [136,137]. For example, researchers from the Washington University School of Medicine created a novel 3D printed human pluripotent stem cells (hPSC)-derived β-cell clusters encapsulation device based on polylactic acid (PLA) and a fibrin hydrogel [138]. After validating good cell viability and insulin production in vitro, such constructs were subcutaneously implanted in mice under immunosuppression. In this study, glucose responsiveness of the implanted cells was demonstrated detecting the according human insulin levels after performing an intraperitoneal glucose tolerance test 12 days after implantation. Moreover, the constructs maintained their structural integrity and were easily retrieved without risk of deformation. Another hybrid bioprinted construct based on polycaprolactone (PCL) and alginate hydrogels was developed to promote quick vascularization after implantation [133]. The construct consisted of a 3D ring-shaped PCL structure with heparinized surface to electrostatically bind VEGF, surrounding an alginate hydrogel core where pancreatic islets were embedded. Thereby, the whole construct was designed to easily implant islets within a mechanically reinforced hydrogel matrix, while actively promoting graft vascularization (Figure 7D). In this study, human pancreatic islet within the bioprinted construct demonstrated to remain viable and functional after the printing process. Additionally, such constructs were implanted in a chicken chorioallantoic membrane (CAM) and vasculogenic potential was confirmed with the observation of new blood vessels formation in the tissue surrounding the graft and even on the surface on the construct (Figure 7E). Other authors followed a different strategy to promote the vascularization at the implantation site using a 3D-printed device [139]. In this case, the 3D construct consisted of a polylactic acid (PLA) disc-shaped device called NICHE, which was coated with platelet-rich plasma (PRP) hydrogel. The PRP coating onto the device was designed to promote vascularization at the implantation site in the subcutaneous space. After vascularization, the chamber of the device was filled with therapeutic cells, thereby providing them a privileged site in the body with high oxygen and nutrient supply. Results demonstrated the highly vasculogenic effect of the implanted device in mice, while providing a favorable environment for long-term cell survival and function. However, additional studies are required to prove the efficacy of this novel vasculogenic cell encapsulation device for pancreatic islets delivery in diabetic animal models [140]. 

### 5.3. Advantages and Limitations

The main advantage of the bioprinting technology is that it offers a unique role in the fabrication of pancreatic tissue-like constructs, through its potential in recreating complex morphologies and multicellular environments. In addition, this technology overcomes the limitations of the conventional islet encapsulation technology, such as the hypoxia state, the lack of vascularization, the diffusion properties of the encapsulation system, etc. [141]. To that end, pancreatic islets could be strategically positioned to mitigate the autoimmune response while enhancing the islets biological function. In this regard, islets may be immunoprotected inside the hydrogel-like bioinks and further inclusion of immunosuppressive or immunomodulatory factors into the bioink formulation may prevent rejection [131]. Additionally, aiming to mimic tissue-specific biological cues, some groups have incorporated decellularized matrices within bioinks, which have been extracted from natural tissues such as adipose, cartilage, and heart [142]. In this sense, including decellularized matrices of pancreas within bioinks, closer biomimetic environments could be achieved to enhance the islet viability and biological function. 

From another point of view, bioprinting also may allow for the fabrication complex constructs with vascular structures surrounding the islets. Multiple print heads could be used for the precise positioning of the bioinks, thus generating an intricate vasculature inside the bioprinted construct to overcome the poor oxygenation and nutrients supply that characterize the islet macroencapsulation [105]. In this regard, sacrificial materials as bioinks, such as pluronic [143] and gelatin [144], in combination with hydrogel-based bioinks have been used in generating these microchannels through inkjet- and extrusion-based bioprinting. To that end, printing sacrificial bioinks, forming precise structures inside the construct, artificial vasculature can be generated after removing the sacrificial material from the construct, thus leaving empty microstructures mimicking an intricate perfusable vascular network [141]. In addition, bioinks may also incorporate endothelial cells and/or slow-releasing compounds, such as VEGF, to promote angiogenesis surrounding the bioprinted structure [133]. Overall, this technology would allow for the embedded islets long-term survival inside the bioprinted graft, while mimicking a functional pancreas. 

However, although the bioprinting approach is a revolutionary technology with high potential for the study and treatment of T1DM, the use of this technique for artificial pancreas fabrication is on the horizon and, for this reason, it still has several limitations due to the early stages of its development. The main technological barrier is that, currently, the choice of bioink materials is limited by stringent printing conditions. Moreover, there are few available standards or commercial bioinks with good biocompatibility and the appropriate biological and physicochemical properties, such as the optimal degree of hydrophilicity, pH neutrality, functional groups, stiffness, elasticity, and porosity, that can mimic the natural pancreatic tissue and achieve the islet physiological function [145]. Additionally, with the current bioprinting technology, human-scale tissues and organs would require too prolonged a time for the printing process, thereby affecting the cell viability of the printed cells [116]. 

However, as printing technology and biomaterial science applied to this technique develop, artificial tissues more similar to organs will be created to finally obtain functional 3D-printed constructs with better therapeutic capacity. 

## 6. Conclusions and Future Perspectives

Islet encapsulation technologies involving nano-, micro-, macroencapsulation, and bioprinting represent promising approaches for T1DM treatment, as they provide the means to transplant islets without immunosuppressive agents and enable the use of alternative islets or β-cells donor sources. However, there are still limitations in each encapsulation modality that hamper their widespread clinical application. The ability to retrieve implanted cells, if needed or desired, is an important biosafety consideration for successful therapy. In this sense, in nano- and microencapsulation approaches, the implanted capsules cannot be contained in a precise location and, consequently, if requiring cell replenishment or in case of graft failure, they cannot be easily and completely removed from the patient, thus supposing a poor degree of biosafety. In contrast, macroencapsulation modality has a high degree of biosafety, as islets are implanted in one single device that can be easily retrieved. However, in these larger configurations, diffusional problems that lead to poor diffusion of oxygen dramatically affect the islets’ viability impeding the long-term functioning of the transplanted islets. In this sense, bioprinting technology is an emerging technology that has the potential to overcome all the mentioned issues by generating artificial pancreatic tissues with vascular structures that would enhance the diffusional issues of macroencapsulation approaches. However, this technology is still in the early stages of development and requires more research in biomaterial science to allow this advanced artificial pancreas fabrication. Overall, advances in biomaterial science, fabrication technologies, safer implantation strategies, angiogenesis inducement, and cell biology, together with progress in regulatory pathways, may allow the translation of these cell encapsulation technologies into medical reality.

## Figures and Tables

**Table 1:** Overview of clinical trials based on transplantation of encapsulated islet.

Investigator or Company	Type of Encapsulation	Hydrogel-like Biomaterial	Islet Source	Implantation Site	Dose	Immunossupresion	Country
Novocell, In. (Viacyte, Inc.) [37]	Nanoencapsulation	PEG	Allogeneic	Subcutaneous	Undetermined	No	USA
Soon-Shiong et al. [38]	Microencapsulation	Alginate-Poly-L-Lysine	Allogeneic	Peritoneum	1040,000 islets equivalents/patient	Yes	USA
Calafiore et al. [39]	Microencapsulation	Alginate-Poly-L-Ornintine	Allogeneic	Peritoneum	400,000–600,000 islets equivalents/patient	No	Italy
Tuch et al. [40]	Microencapsulation	Barium cross-linked alginate	Allogeneic	Peritoneum	178,000 islets equivalents/ patient	No	Australia
Living Cell Technologies (LCT) [41]	Microencapsulation	Alginate-Poly-L-Ornintine	Xenogeneic-porcine insulin-producing cells	Peritoneum	5000–10,000 islets equivalents/kg body weight	No	Australia
Dufrane et al. [42]	Macroencapsulation-Monolayer Cellular Device	Collagen/Alginate	Allogeneic	Subcutaneous space	Undetermined	Undetermined	Belgium
Beta-O2 Technologies [43]	Macroencapsulation-β-Air device	Alginate	Allogeneic	Peritoneum	2100 islets equivalents/kg body weight	No	Germany
Alejandro Rodolfo et al. [44]	Macroencapsulation-BioHub	Thrombin/patient’s own plasma	Allogeneic	Omentum	600,000 islets equivalents/patient	Yes	USA
